# Reports of Cases

**Published:** 1898-12

**Authors:** 


### REPORTS OF CASES.

### CLINICAL REMARKS ON CONTRACTION OF THE SOLE.[1]

By S. J. J. Harger, V.M.D.,
VETERINARY DEPARTMENT, UNIVERSITY OF PENNSYLVANIA.

Contraction of the horny sole of the foot is a rather rare deformity, and is, without doubt, often diagnosed as some other obscure and chronic lameness of the foot.

The animal, a gray gelding, was brought to the veterinary hospital ten weeks ago as an out-patient. Had for a month been quite lame in the off, and slightly so in the near, foot.

*Symptoms.* The symptoms were well-defined. The lameness in the off foot was very marked and presented the characters of foot lameness, "supporting leg" lameness. Examination, outside of the foot, was negative. The deformity of the foot was characteristic. The surface of the wall, instead of being straight from above to below, was convex, especially toward the lower border; the lower edge of the wall appeared as if curled in toward the bottom of the foot, and the circumference of the wall directly at the plantar border was less than at a point a short distance above; the heels were contracted and curved inward. When the foot was lifted from the ground the sole was found to be very concave and pushed upward against the bottom of the foot. The wall and sole were thick and strong; the frog moderately developed. Pressure with the pincers over the centre of the sole produced slight pain; this is often absent. Sometimes there is pulsation of the digital artery and a blood-stained aspect of the white line.

Instead of affecting the entire foot, as in this case, the contraction may affect only certain regions of the wall and sole, especially the toe and laminæ.

The shrinkage of the wall being especially toward its lower border and the antero-posterior and lateral diameters of the bottom of the foot thus decreased, the sole can give way in only one direction—arching upward; hence the excessive concavity and the pain by the compression of the velvety tissue between the sole and the third phalanx. This fact suggests a very peculiar phenomenon in the anatomo-physiologic arrangement of the foot. The velvety tissue, although intimately packed in between the bottom of the

[1] Abstract of Clinical Lecture.

third phalanx and the horny sole, is not subjected to any appreciable pressure; that is, there is not sufficient downward pressure by the coffin-bone against the superior face of the sole to produce in the normal state pain from compression of this sensitive tissue. The essential means of attachment of the third phalanx, that which prevents this bone from being displaced downward in any appreciable degree, is the intimate union between the fleshy and the horny laminæ on the inside of the wall; in other words, the third phalanx is firmly maintained in its position by its suspension from the inside of the wall.

It is true that the third phalanx slightly rotates downward, with the toe as the fulcrum, and that the sole is depressed, flattened when the animal's weight is on the foot, but the latter phenomenon, the lowering of the sole, is due essentially to the descending movements of the frog which, by its intimate union with the sole, drags the latter along. If the latter action is prevented by excessive thickness or concavity of the sole, the pressure of the shoe or a stone wedged in between the shoe and the frog, great pain and lameness quickly result. The frog, by its intimate contiguity with the bars and sole, is indirectly limited in its downward movements by the attachment of the periphery of the sole to the white line, as well as by its own attachment to the bars and heels. This mechanism also assists the suspension of the third phalanx in supporting the weight on the member.

*Diagnosis.* The diagnosis was based upon the concavity of the sole, the deformity of the wall, and pain on pressure on the centre of the sole. Contraction of the sole may be confounded with ulceration in the last inter-phalangeal articulation and with navicular disease. In the first case we must rely upon the absence of the characteristic deformity of the hoof described above. In navicular arthritis we would consider the history of the case, the intermittent and "warming out" lameness, absence of pulsation of the collateral artery of the cannon, and the differences between ordinary lateral contraction of the wall accompanying navicular disease and that of contraction of the sole. It is possible that the two conditions may exist simultaneously.

*Prognosis.* This is favorable, but some time is necessary to effect a recovery.

*Treatment.* This consists principally in hygiene of the foot and shoeing calculated to expand the wall and lower the sole. This is very essential.

In the animal in question the feet were soaked two or three times weekly to render the horn soft, and a hoof ointment applied *as soon as* the hoof was dry.

The wall was pared down as much as was permissible, and the sole thinned as much as possible. The sole may be pared until it yields to the pressure of the finger.

A plain shoe, with bevelled toe, leather and oakum, was applied by the hospital farrier. The bearing surface of the shoe, instead of being horizontal, was slightly bevelled outward in order to assist in the expansion of the wall. The shoes were reset every two weeks.

At present the animal shows a trifling lameness in the right foot. He has been driven every day, and his ultimate recovery is expected. In fact, this would probably be the case now had the animal been returned more often and the treatment strictly carried out as recommended.

---

### TREATMENT OF OPEN JOINT.[1]

By J. C. Michener, V.S.,
COLMAR, PA.

Having reported a few cases of open-joint treated with an antiseptic blister, I would like to add one more. On the 11th of last August, when a high-spirited horse belonging to Mr. A. C. Haldeman, of Line Lexington, was being bedded by his twelve-year-old son, the boy pricked the horse at the fetlock. The horse made a bound forward and gave a violent kick, running the prong into the postero-exterior part of the thigh. The end of the handle striking in the corner of the stall, the fork remaining in, the horse continued kicking and surging about until the handle was broken into pieces and the prong driven in its full length. It came through on the inside of the joint, just beneath the patella. Great excitement in the village; the horse squealing, kicking, throwing himself down, and the blood flying all around. After getting the horse somewhat quieted and secured, a stalwart fellow pulled out the fork, having to make the second effort and use his full strength. The prong was bent in different places and directions.

On arriving, two hours later, found an ugly lacerated wound on the outside of thigh, three inches wide and into the bone and having a pocket three inches deep. Inside of joint showed a small

[1] Read before the semi-annual meeting of the Pennsylvania State Veterinary Medical Association, Pittsburg, September, 1898.

puncture where point of prong came through. The horse was suffering intensely, keeping the injured limb in constant motion.

After cleansing wound and limb, I rubbed in blister-ointment thoroughly over the entire joint, and also plastered the exterior wound full of the same (getting severely criticised for so doing). Next morning horse stood firmly upon the injured limb, breathing naturally and feeding well. Rubbed on more ointment, although the blister was acting freely and the leg already enormously swollen. The swelling kept very tense for three days, but the horse stood and tread firmly. Gave him much walking exercise (for which I was again severely criticised). On the fourth day the outside wound commenced to discharge a healthy pus. It was syringed twice daily with chloride-of-zinc lotion, ten grains to the ounce, and the whole leg bathed frequently with cold vinegar and water, equal parts. Upon the twelfth day the leg had resumed its natural size, and upon the nineteenth day the horse was put to work, the wound having healed and lameness gone.

In justice to some of the Dutch villagers, will state that the fork was carefully greased and put in a dry place *before I arrived.*

---

Berlin has the smallest elephant in the world. It is thirty-nine inches high and weighs 160 pounds.

The smallest horse in the world is a Shetland pony owned by the Marquis of Carcano. Its height does not exceed twenty-eight inches.

Waste of Human Life. While progress in civilization has brought greater care of human life, there is yet a prodigal waste. Dr. A. Hill, Vice-Chancellor of Cambridge University, states that one-fourth of all the diseases that destroy life are absolutely preventable, and that if the practice of hygiene were only on a level with its theory the average longevity would be raised at once from fifty to fifty-five years. The greater number of diseases over which the individual has control are due to mistakes in eating and drinking. One purpose yet to attain is a more exact knowledge by every citizen of the causes and properties of preventable diseases, but it is hardly surprising that the knowledge is still so slight, when even medical men hardly realized the contagious character of consumption twenty years ago, although one-third of the cows in England were tuberculous and half the milk sold distributed the bacillus of tuberculosis.

